# Smurf1 regulates ameloblast polarization by ubiquitination‐mediated degradation of RhoA

**DOI:** 10.1111/cpr.13387

**Published:** 2022-12-29

**Authors:** Haoman Niu, Fei Bi, Wenjun Zhao, Yuchan Xu, Qi Han, Weihua Guo, Yu Chen

**Affiliations:** ^1^ State Key Laboratory of Oral Diseases, West China Hospital of Stomatology Sichuan University Chengdu Sichuan China; ^2^ Department of Oral Pathology, West China School of Stomatology Sichuan University Chengdu Sichuan China; ^3^ Department of Pediatric Dentistry, West China School of Stomatology Sichuan University Chengdu Sichuan China

## Abstract

Cell polarity is essential for ameloblast differentiation and enamel formation. Smurf1 can mediate cell polarization through ubiquitination degradation of specific substrates. But it remains unclear whether Smurf1 could regulate ameloblast polarity and the underlying mechanism. Here, immuno‐fluorescence staining and RT‐qPCR were applied to detect the expression of Smurf1 and F‐actin. A mouse lower incisor defect model was constructed. Scanning electron microscope, rat lower incisor culture, western blot, wound healing assay and trans‐well migration assay were performed to detect the influence of Smurf1 knockdown on ameloblast. IF double staining, western blot and co‐immunoprecipitation were conducted to detect the interaction between Smurf1 and RhoA. The in vivo experiment was also performed. We found that Smurf1 was mainly expressed in the membrane and cell cortex of ameloblast, similar to F‐actin. Smurf1 expression increased along ameloblast polarization and differentiation. After knocking down Smurf1, the cytoskeleton and cell morphology changed and the cell polarity was damaged. Smurf1 regulated ameloblast polarity through ubiquitination degradation of activated RhoA in vitro. Local knockdown of Smurf1 in rat lower incisor ameloblast resulted in ameloblast polarity loss, enamel matrix secretion disorder and chalky enamel, but RhoA inhibitor Y‐27632 could reverse this effect. Collectively, Smurf1 could regulate the polarization of ameloblast through ubiquitination degradation of activated RhoA, which contributed to the knowledge of tooth development and provided new research ideas for cell polarity.

## Graphical abstract

Smurf1 positively expressed in the dental epithelium of enamel organ and lower incisor, and its expression level increased along the differentiation maturation of ameloblast. Knocking down Smurf1 in HAT‐7 cells resulted in expression location changes of Smurf1, cell morphology alteration and cell polarity loss. Smurf1 degraded activated RhoA in a ubiquitination‐proteinase pathway. In vivo, knockdown of Smurf1 in rat lower incisors' ameloblasts led to chalky enamel, ameloblast polarity disruption and enamel matrix secretion disorder, but RhoA inhibitor Y‐27632 could reverse this effect.

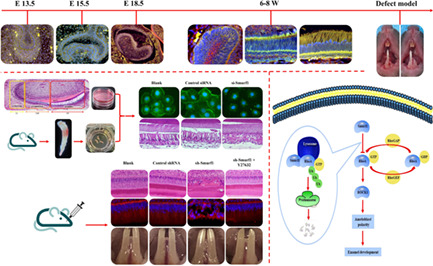

## 1 INTRODUCTION

Ameloblasts (AB) are the key cells in tooth enamel formation. These cells are of epithelial origin and are involved in the whole process of enamel formation, including the synthesis and secretion of enamel matrix protein, the reabsorption and degradation of enamel matrix protein and the transport of calcium salts.^1^ Abnormal interference in the differentiation and maturation of AB can lead to diseases related to enamel development such as enamel hypoplasia and enamel hypomineralization,^2^ thus affecting patients' mastication, pronunciation and aesthetics. Cell polarity is crucial to the differentiation and maturation of AB and the formation of enamel.

Cell polarity refers to the asymmetric distribution of cytoskeleton, organelles and biomacromolecules due to the cooperation or rejection of polarity protein complexes,^3^ so that different regions of the cell can perform different functions, which plays an important role in embryonic development,^4^ cell differentiation,^5^ cell migration,^6^ asymmetric cell division (ACD)^7^ and tumour development.^8^ According to the distribution characteristics of polarity protein complexes and cell morphology, cell polarity can be divided into four categories: Apical‐basal polarity (ABP),^9^, ^10^ Planar cell polarity (PCP),^11^, ^12^ Front‐rear polarity (FRP)^13^, ^14^, ^15^ and ACD.^16^, ^17^, ^18^ ABP is the one category of cell polarity which closely relates to the differentiation and maturation of AB and the formation of enamel. The AB in terminal differentiation and mature condition show a typical ABP structure, which is mainly manifested in the following aspects: the cells being high columnar and the nuclei being far away from the basement membrane and arranged in a palisade pattern. In addition, there are many clusters of rough endoplasmic reticulum near the basement membrane and parallel to the long axis of the cell. The Golgi complexes are developed well. Tomes' processes are formed at the distal end of the cell, which contributes to the preparation of enamel matrix secretion. Whereas, the precursor cell of ameloblast, pre‐ameloblast (PAB) appears non‐polarized with the structural characteristics of short columnar, large and centre‐sited nucleus, mitochondria and Golgi complex scattered in the cytoplasm and immature rough endoplasmic reticulum. Above elaboration indicates that in the process of cell polarity formation, differentiation and maturation, the morphological characteristics, organelle distribution and function of AB have significantly changed.

Smad ubiquitination regulatory factor 1 (Smurf1), a ubiquitin‐protein ligases belonging to the ubiquitin‐proteasome system (UPS), has been found to regulate cell polarity for ubiquitination degradation of specific substrates through mediating the binding between Ubiquitin (Ub) and protein substrates via its C‐terminal HECT domain.^19^, ^20^ In addition to cell polarity regulation,^21^ Smurf1 has also been found to play an important role in a series of biological processes such as cell adhesion and migration,^22^, ^23^, ^24^ nerve axon growth,^25^ bone regeneration and bone homeostasis,^26^, ^27^ and autophagy.^28^ Among the Smurf1 substrates found so far, RhoA, belonging to the Rho GTPases family, is involved in regulating cell polarity,^29^ cell migration,^6^ cell differentiation,^30^ and tumour development.^31^ RhoA is also essential during the odontogenesis process.^32^ The expression of Rho GTPases is regulated by post‐translational modifications, such as lipid modification, phosphorylation and ubiquitination.^33^ Particularly, in the ubiquitination process, Smurf1 can catalyse the ubiquitination degradation of RhoA in activated state.^34^, ^35^

**FIGURE 1:**
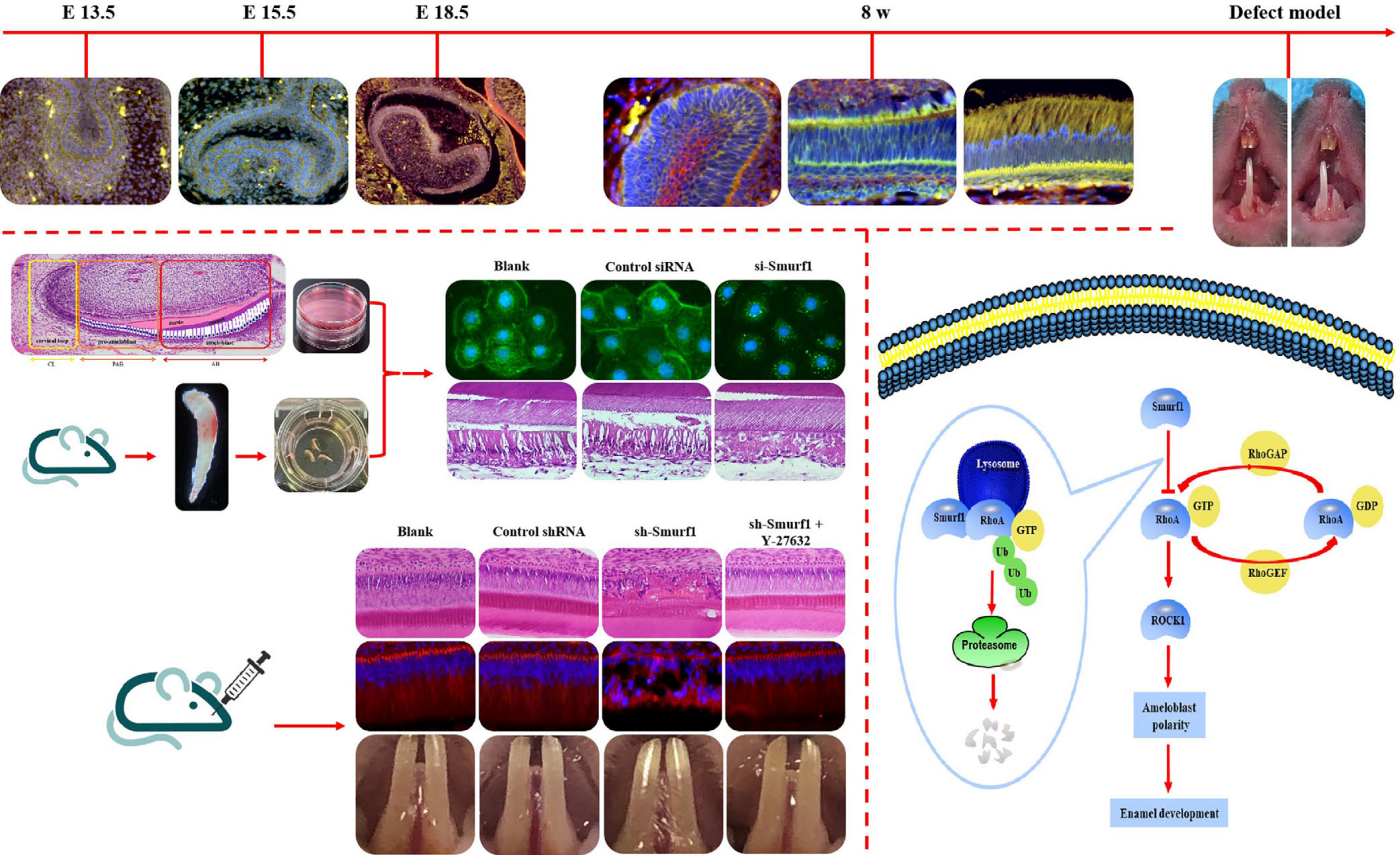
Graphical abstract

Smurf1 can mediate cell polarization through ubiquitination degradation of specific substrates, but whether Smurf1 is involved in AB polarity has not been reported. RhoA is one of the substrates of Smurf1 and also plays an important role in the polarity of AB. However, it remains unclear whether Smurf1 can regulate ameloblast polarity by degrading RhoA during tooth development. Therefore, we conducted in vitro and in vivo experiments to study the role of Smurf1 in regulating AB polarity during cell differentiation and the underlying mechanism specifically in a RhoA ubiquitination‐mediated degradation way (Figure 1).

## 2 MATERIALS AND METHODS

### 2.1 Animals and tissue sections

The C57BL/6 mice and Sprague–Dawley (SD) rats were purchased from the experimental Animal Laboratory of Sichuan University. All animal experiments were approved by the Ethics Committee of the State Key Laboratory of Oral Diseases, West China Hospital of Stomatology (Chengdu, Sichuan). The mandibles were dissected from mice on embryonic day 13.5 (E13.5), E15.5, E18.5, and postnatal (PN) week 8 (8 w), fixed in 4% paraformaldehyde overnight, demineralized with 10% EDTA (pH 8.0), dehydrated, embedded in paraffin wax and serially sectioned at 5 μm for future experiments.

### 2.2 Ameloblasts' isolation

Carefully remove the surrounding bone tissues and acquire mandibular incisor of the adult mice under the stereo‐microscope. Immerse the incisors into 2% collagenase type I solution at 37°C for 45 min and gently shake the container every 5 min to separate attached mesenchymal tissues from dental epithelium as much as possible. Carefully peel off the surrounding mesenchymal tissues and divide the mandibular incisor into three parts according to the anatomical points (Figure S1A) under the stereo‐microscope. The enlarged tail of the epithelium ends belonged to the cervical loop (CL) group. The epithelial tissues from the enamel deposition point to the crown side belonged to the AB group. Remaining tissues sited in the middle of CL and AB group belonged to the PAB group.^36^

### 2.3 Immunofluorescence staining

Tissue sections and HAT‐7 (Osaka Dental University) cells were fixed, washed, blocked and incubated with antibodies as follows: Smurf1 (1:50, Santa Cruz, USA), F‐actin (Phalloidin‐iFluor 488, 1:1000, Abcam, USA), F‐actin (Phalloidin‐iFluor 594, 1:1000, Abcam, USA), RhoA (1:50, Proteintech, China), AMBN (1:50, Santa Cruz, USA), AMGN (1:50, Santa Cruz, USA), Alexa Fluor 594 goat anti rabbit (1:1000, Invitrogen, USA), Alexa Fluor 594 goat anti mouse (1:1000, Invitrogen, USA) and Alexa Fluor 488 goat anti mouse (1:1000, Invitrogen, USA). The images were observed and taken under fluorescence microscope (Olympus, Japan).

### 2.4 Quantitative real‐time PCR analysis **(RT‐qPCR)**

The total RNA was extracted from tissues and cells using Trizol (Invitrogen, USA). The cDNA was synthesized by the Prime Script™ RT‐PCR with gDNA Eraser Kit (TaKaRa, Japan). The SYBR® Premix Ex Taq II (TaKaRa, Japan) was applied in the RT‐qPCR tests. The reaction was performed with QuantStudio 3 Flex (Applied Biosystems, USA). The primer sequences were listed in Table 1.

**TABLE 1:** The primer sequences of qRT‐PCR

Gene	Sequence
GAPDH (mouse)	
Forward	AAGAAGGTGGTGAAGCAGGCATC
Reverse	CGGCATCGAAGGTGGAAGAGTG
Ameloblastin (mouse)	
Forward	ACAACGCATGGCGTTTCCAA
Reverse	ACCTTCACTGCGGAAGGATA
Amelogenin (mouse)	
Forward	TTCAGCCTCATCACCACCTT
Reverse	AGGGATGTTTGGCTGATGGT
Ki‐67 (mouse)	
Forward	TTTCAGGTCTCTGGAAGCAGTCA
Reverse	ATCTCCATAATTGCTTTGATTGCA
Smurf1 (mouse)	
Forward	AGCATCAAGATCCGTCTGACA
Reverse	CCAGAGCCGTCCACAACAAT
GAPDH (rat)	
Forward	GTGCTGAGTATGTCGTGGAGTCT
Reverse	ACAGTCTTCTGAGTGGCAGTGA
Smurf1 (rat)	
Forward	CCGTGGAGTGAAGAGCA
Reverse	GAGGGAGACGAGCCTTTT
RhoA (rat)	
Forward	GACCAGTTCCCAGAGGTTT
Reverse	CTGTGTCCCATAAAGCCAA

### 2.5 Mouse lower incisor defect model

The left lower incisors of 8‐week‐old mice were grinded off with the dental emery drill. The right lower incisors were used for self‐control. Three days after the operation, the mice were executed and the teeth were observed under natural light. The dental epitheliums were isolated by CL, AB and PAB group as previously described.

### 2.6 HAT‐7 cells' transfection

The rat AB cell line namely HAT‐7 cells were cultured with DMEM/F‐12 (Gibco, USA) supplemented with 10% fetal bovine serum (FBS, Gibco, USA) and 1% penicillin–streptomycin at 37°C with 5% CO_2_. The medium was changed every 2 days. The cells were seeded into 6‐well plate. Once achieving 70% confluence, the cells were transfected with the following agents: the control siRNA, Smurf1 siRNA1, Smurf1 siRNA2, Smurf1 siRNA3, vector, Smurf1 overexpression plasmid, wild‐type RhoA overexpression plasmid (RhoA WT), the T19N sustained inhibitory plasmid (RhoA T19N) and the RhoA G14V sustained activation plasmid (RhoA G14V) (Yeda, China). The sequences of the siRNAs were shown in Table 2. The proteasome inhibitor, MG132, was added at a concentration of 25 μM 6 h before collecting cells.

**TABLE 2:** The sequences of siRNAs

Group	Sequence
Control siRNA	5′‐UUCUC GAACGUGUCACGUTT‐3′
	3′‐ACGUGACACGUUCGGAGAATT‐5′
Smurf1 siRNA1	5′‐CACAUCAUGAAUCACCAGUTT‐3′
	3′‐ACUGGUGAUUCAUGAUGUGTT‐5′
Smurf1 siRNA2	5′‐GAAACCCAAUGGCAGAAAUTT‐3′
	3′‐AUUUCUGCCAUUGGGUUUCTT‐5′
Smurf1 siRNA3	5′‐GGAGGUUUAUGAGAGGAAUTT‐3′
	3′‐AUUCCUCUCAUAAACCUCCTT‐5′

### 2.7 Western blot

Cells were lysed with RIPA buffer (Millipore, USA). Extracted proteins were quantified using the BCA Protein Assay (Bio‐Rad, USA). 20 μg of protein lysates from each group were separated by SDS‐polyacrylamide gel electrophoresis (SDS‐PAGE) then transferred onto polyvinylidene fluoride (PVDF) membranes. After being blocked with 5% skim milk, the PVDF membranes were incubated with antibodies. The primary ones were as follows: Smurf1 (1:200, Santa Cruz, USA), AMBN (1:500, Santa Cruz, USA), AMGN (1:500, Santa Cruz, USA), MMP20 (1:1000, Proteintech, China), KLK4 (1:500, Absin, China), RhoA (1:1000, Proteintech, China) and GAPDH (1:1000, Servicebio, China).

### 2.8 Scanning electron microscope

HAT‐7 cells were seeded into 12‐well plates, and the experiment was carried out when the cells grew to about 25% confluence. Rinse the AB with phosphate buffered solution (PBS) and fix the cells with 3% glutaraldehyde solution at 4°C for 2 h. Rinse the AB with PBS for 10 min, twice. Fix the cells with 1% osmic acid at 4°C for 1 h. Rinse the AB with PBS for 10 min, twice. Dehydrate cells with graded ethanol. Dry the cells and sputter them with gold to render electroconductiveness. Observe the treated AB with a SEM and collect images.

### 2.9 In vitro rat lower incisor culture

The postnatal 5–7 days rat lower incisors were isolated and cultured in trans‐well chambers of 6‐well plate in DMEM/F‐12, supplemented with 10% FBS, 1% penicillin–streptomycin and 200 mg/l ascorbic acid for 7 days. The medium was changed every 2 days. Collected tissues were fixed in 4% paraformaldehyde, and sliced for further H&E staining.

### 2.10 Wound healing assay and trans‐well migration assay

In the wound healing experiment, the migration area of cells was observed at 0 and 24 h under light scope and measured (Image J, USA). The trans‐well chambers with 4 μm pores (Corning, USA) were placed in a 24‐well plate for the migration assay. The HAT‐7 cells resuspended with medium containing 1% BSA were seeded into the upper chambers. The lower chambers were filled with complete medium. After 24 h incubation, the cells that migrated through the pores were stained with Giemsa and observed using a light microscope.

### 2.11 Cell proliferation

A cell count kit‐8 (CCK‐8, Dojindo, Japan) was used to quantitatively evaluate the proliferation ability of HAT‐7 cells. The cells were seeded in 96‐well plate with an initial density of 3000 cells per well. Detections were launched at 0, 24, 48, 72 and 96 h with six parallel replicates. After incubation with freshly prepared medium containing 10% CCK‐8 solution at 37°C for 1 h, the absorbance of the supernatant was detected at 450 nm using a spectrophotometer.

### 2.12 Apoptosis analysis

After being washed with PBS, HAT‐7 cells were digested with 0.25% trypsin without EDTA, centrifuged at 1000 r/min for 5 min and washed with PBS. Half of the cells were added with Annexin V‐FITC and PI using Annexin V‐PE/7AAD apoptosis detection kit (KeyGEN, China) following the manufacturer's protocol. The remaining cells were divided equally into three tubes for blank control, single staining control of Annexin V‐FITC, and single staining control of PI. After incubation with the antibodies, the cells were detected with flow cytometer. The data was analysed in FlowJo software.

### 2.13 Co‐immunoprecipitation

HAT‐7 cells were washed twice with pre‐cooled PBS, treated with 500 μl lysis buffer, split three times with ultrasonic at 5% power for 5 s, and centrifuged for 5 min at 4°C with 15,000 r/min. The supernatant was collected, and 40 μl lysate was taken as input and boiled with 10 μl 5× loading buffer for 10 min. The remaining lysate was added with the antibody of Smurf1 and mixed overnight at 4°C. On the second day, 20 μl A/G‐agarose was added into the samples and mixed overnight at 4°C to drag the antibody. On the third day, the A/G‐agarose was washed with lysis buffer for five times. At the last washing, the A/G‐agarose was washed with the solution of 40 μl lysis buffer and 10 μl 5× loading buffer. Subsequently, the solution was boiled at 100°C for 10 min. The samples went through Western blot analysis with the same antibodies used above.

### 2.14 In vivo experiment

The PN 3d rats were divided into four groups (*n* = 5) randomly and injected with normal saline, lentivirus packed with control shRNA, sh‐Smurf1 and mixture of sh‐Smurf1 and Y‐27632 at the mandible adjacent to the dental epithelium on PN 3d and PN 17d. The mandibles were collected on PN 25d. After fixed with 4% paraformaldehyde overnight, the mandibles were demineralized with 10% EDTA (pH 8.0) and made into slices for H&E staining and IF staining.

### 2.15 Statistical analysis

All experiments were performed independently at least three times. The statistical analysis was carried out in SPSS 21.0 software using Student's *t*‐test or one‐way ANOVA. *p* < 0.05 was considered statistically significant.

## 3 RESULTS

### 3.1 Expression of Smurf1 during ameloblast differentiation

**FIGURE 2:**
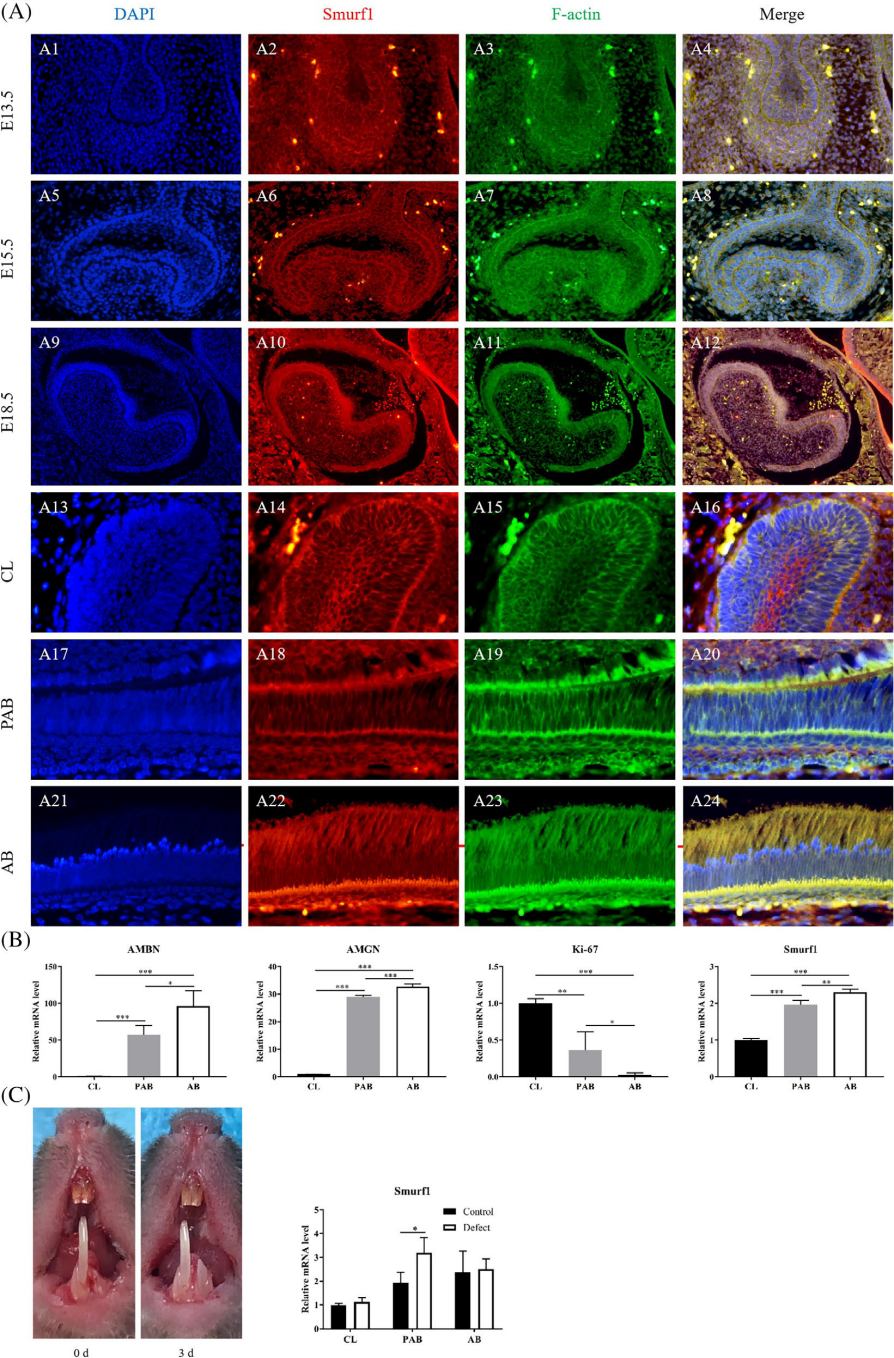
Expression of Smurf1 during ameloblast differentiation. (A) Smurf1 and F‐actin expression on E13.5 (A1–A4), E15.5 (A5–A8) and E18.5 (A9–A12); Smurf1 and F‐actin expression on 8 weeks of different sites–CL (A13–A16), PAB (A17–A20), and AB (A21–A24) [magnification: 400×]. (B) Expression of ameloblastin (AMBN), amelogenin (AMGN), ki‐67 and Smurf1 of different regions of dental epitheliums (CL, PAB and AB) by RT‐qPCR. (C) Conduction of the defect left lower incisor of mice and expression of Smurf1 in different regions of dental epitheliums (CL, PAB and AB) in injury‐repair process by RT‐qPCR

The enamel organ displayed three major stages during odontogenesis with distinguished morphologies and functions, namely bud stage (E13.5), cap stage (E15.5) and bell stage (E18.5) (Figure S1B). Smurf1 was positively expressed in all stages of the enamel organ, mainly located in cell membrane and cell cortex (Figure 2A, A1–A12). The expression position of Smurf1 was basically consistent with that of F‐actin.

The dental epitheliums of lower incisors of mice at 8 w could be divided into three groups of CL, PAB and AB according to their position and cell morphology (Figure S1C). In the CL region, Smurf1 strongly expressed on the cell membrane and the cell cortex of the inner enamel epithelium, the outer enamel epithelium and the stellate reticulum (Figure 2A, A13–A16). In the short columnar PAB district, Smurf1 mainly expressed on the cell membrane and the cell cortex, especially at the apical and basal regions (Figure 2A, A17–A20). In the columnar AB area, apart from highly expressed in the apical and basal regions, Smurf1 was also positively expressed in most of the cytoplasm on the basement membrane side. In addition, the expression pattern of Smurf1 was similar to F‐actin (Figure 2A, A21–A24). These findings suggested a strong association between Smurf1 and F‐actin, and indicated that Smurf1 might participate in the differentiation and polarization of AB.

RT‐qPCR showed that as AB maturing from CL to AB, the expression of differentiation marker ameloblastin (AMBN) and amelogenin (AMGN) increased, and the expression of proliferation marker Ki‐67 decreased (Figure 2B, *p* < 0.05). These results suggested that three groups of dental epitheliums were successfully dissected and acquired. In the meantime, the expression level of Smurf1 significantly increased along AB maturing from CL to AB (Figure 2B, *p* < 0.05). This suggested that Smurf1 could be participating in the differentiation and polarization process of AB.

Three days after the operation of grinding off the left lower incisors of mice, we discovered new enamel formation. Compared to the control side, the expression level of Smurf1 at the defected side was higher in PAB group (Figure 2C, *p* < 0.05). These outcomes indicated PAB cells go through certain process during repair involving Smurf1 in.

### 3.2 The influence of Smurf1 knockdown on ameloblast polarization

**FIGURE 3:**
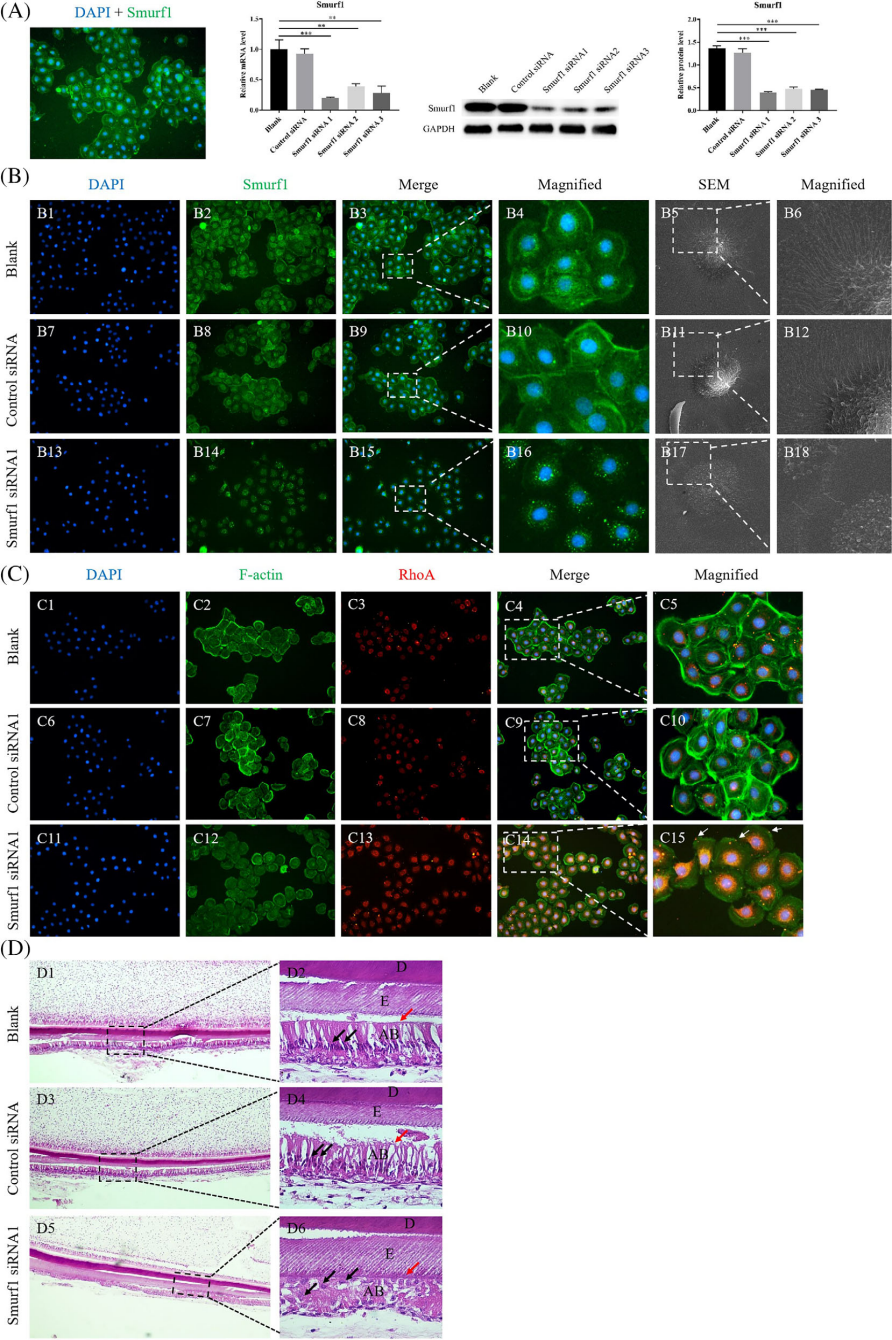
Knockdown Smurf1 affects the ameloblast polarity. (A) Immuno‐fluorescence of Smurf1 in HAT‐7 cells [magnification: 100×]; Smurf1 knockdown efficiency detected by RT‐qPCR; Smurf1 knockdown efficiency detected by western blot and quantitative analysis. (B) Immuno‐fluorescence of Smurf1 in HAT‐7 cells treated with blank, control siRNA and Smurf1 siRNA1 (B1–B4, B7–B10, B13–B16) [magnification: B1–B3, B7–B9, B13–B15, 100×; B4, B10, B16, 400×]; 3D morphology detection of HAT‐7 cells treated with blank, control siRNA and Smurf1 siRNA1 by scanning electron microscope (B5, B6, B11, B12, B17, B18) [magnification: B5, B11, B17, 5000×; B6, B12, B18, 10,000×]. (C) Immuno‐fluorescence of F‐actin and RhoA in HAT‐7 cells treated with blank, control siRNA and Smurf1 siRNA1 [magnification: C1–C4, C6‐C9, C11–C14, 100×; C5, C10, C15, 400×]. (D) H&E staining of lower incisors of postnatal 5–7 days SD rat cultured in a 3D in vitro environment of trans‐well membrane system with blank, control siRNA and Smurf1 siRNA1 [magnification: D1, D3, D5, 40×; D2, D4, D6, 400×]

In HAT‐7 cells, Smurf1 mainly expressed in the membrane and nucleus, with a little in the cytoplasm around the nucleus. Smurf1 siRNA1 could inhibit 82% expression at RNA level and 71% expression at protein level (Figure 3A, *p* < 0.05). Smurf1 siRNA1 showed the highest interference efficiency and was selected for subsequent experiments.

The fluorescence intensity of Smurf1 expression decreased in Smurf1 siRNA1 group. Simultaneously, the expression site of Smurf1 changed from cell membrane to perinuclear cytoplasm (Figure 3B, B4, B10, B16). The results of SEM showed that in the Smurf1 siRNA1 group, cells were collapsed and the microvilli disappeared. Only a couple of residual and broken microvilli could be seen around the cells (Figure 3B, B17–B18). In Smurf1 knockdown cells, the expression of F‐actin in the membrane and the cell cortex significantly decreased, yet increased in the cytoplasm. Furthermore, in Smurf1 siRNA1 group, RhoA's expression in the perinuclear cytoplasmic region increased, and spotty positive expression was observed on the membrane (Figure 3C). These outcomes suggested that Smurf1 knockdown resulted in the alteration of expression sites of itself, the decreased expression level of Smurf1 and F‐actin and increased expression level of RhoA. Furthermore, the cell morphology was significantly affected due to Smurf1 deficiency.

After seven‐day culture of lower incisors of PN 5‐7d SD rat in a trans‐well membrane system (Figure S1D, D1–D5), samples were collected and performed with H&E staining. In the blank group and the negative control group, the AB arranged by high columnar shape, and the nuclei were far away from the basement membrane (Figure 3D, D2, D4), which was consistent with the morphology of the normal incisor. However, in the Smurf1 siRNA1 group, the AB were disordered and the polarity structure was damaged with elliptic nuclei (Figure 3D, D6). These results demonstrated that Smurf1 knockdown could affect the ameloblast polarity in 3D environment.

### 3.3 The influence of Smurf1 knockdown on ameloblast differentiation, migration, proliferation **and apoptosis**

**FIGURE 4:**
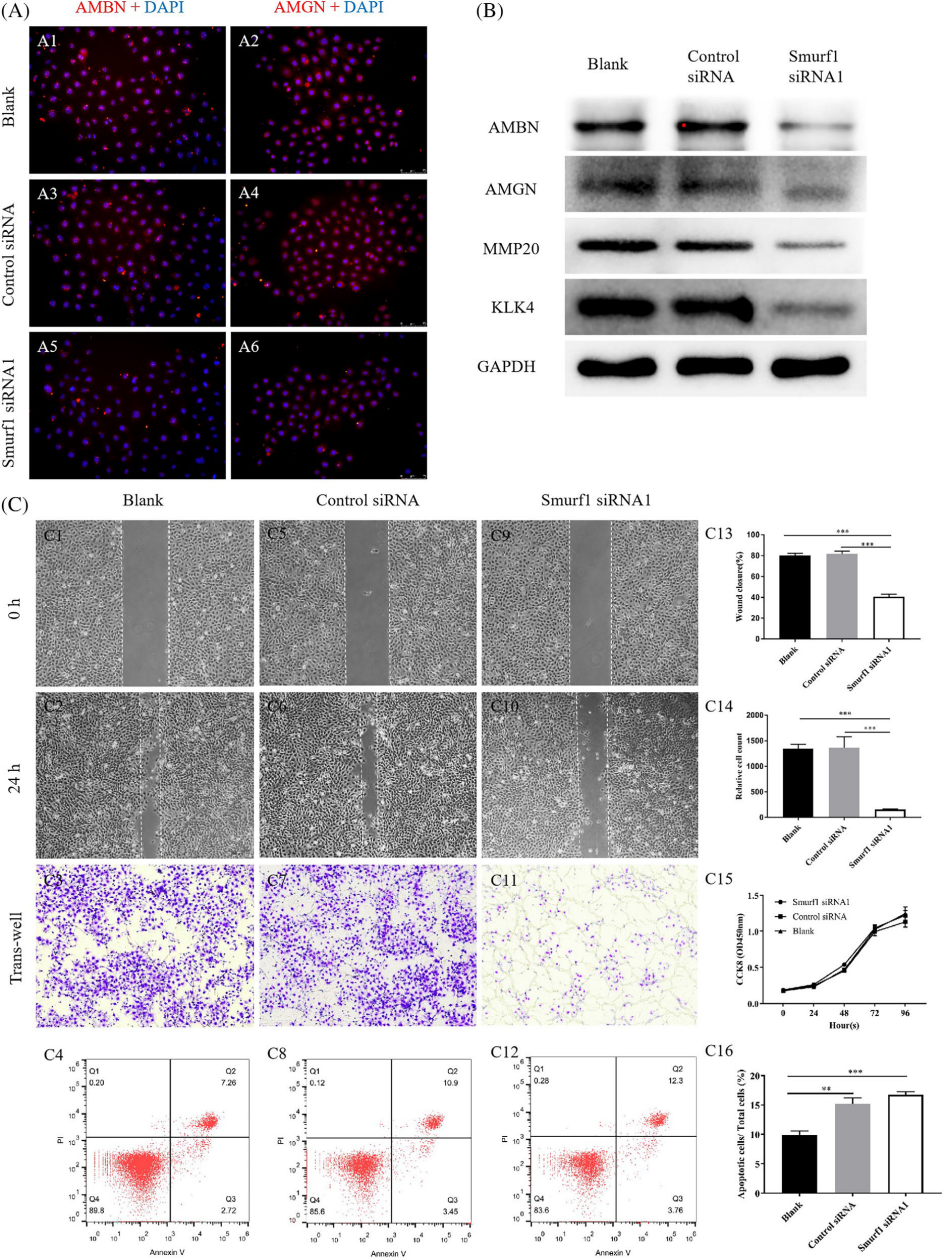
Knockdown Smurf1 affects other biological behaviours of ameloblast. (A) Immuno‐fluorescence of AMBN and AMGN in HAT‐7 cells treated with blank, control siRNA and Smurf1 siRNA1 [magnification: 100×]. (B) Protein expression detection of AMBN, AMGN, MMP20 and KLK4 in HAT‐7 cells treated with blank, control siRNA and Smurf1 siRNA1 by western blot. (C) Migration ability test of HAT‐7 cells treated with blank, control siRNA and Smurf1 siRNA1 by wound healing assay (C1, C2, C5, C6, C9, C10) [magnification: 100×] and its quantitative analysis (C13); Migration ability test of HAT‐7 cells treated with blank, control siRNA and Smurf1 siRNA1 by trans‐well assay (C3, C7, C11) [magnification: 100×] and its quantitative analysis (C14); Cell proliferation test of HAT‐7 cells treated with blank, control siRNA and Smurf1 siRNA1 by CCK8 assay (C15); Cell apoptosis test of HAT‐7 cells treated with blank, control siRNA and Smurf1 siRNA1 by flow cytometry (C4, C8, C12) and its quantitative analysis (C16)

The expression of AMBN and AMGN were significantly decreased after Smurf1 knockdown testified by IF staining (Figure 4A). Western blot results showed that the expression of AMBN, AMGN, MMP20 and KLK4 were significantly reduced in Smurf1 siRNA1 group (Figure 4B). These results suggested that Smurf1 knockdown could inhibit the differentiation of AB.

The scratch test showed that the healing area in Smurf1 knockdown group was the smallest (Figure 4C, C13, *p* < 0.05). The trans‐well experiment showed that the number of cells passing through the chamber to the lower layer significantly reduced in the Smurf1 siRNA1 group (Figure 4C, C14, *p* < 0.05). These results indicated that Smurf1 knockdown could inhibit ameloblast migration.

CCK8 test showed that there was no significant difference in the absorbance values of each group within 96 h after transfection (Figure 4C, C15, *p* > 0.05). Flow cytometry results showed that compared with the blank control group, the proportion of apoptosis cells in negative control group and Smurf1 siRNA1 group increased. But the difference between the negative control and Smurf1 siRNA1 group had no statistical significance (Figure 4C, C16, *p* > 0.05). These results suggested that Smurf1 knockdown had no effect on ameloblast proliferation and apoptosis.

### 3.4 The mechanism of Smurf1 affecting ameloblast polarization by ubiquitination‐mediated degradation of RhoA

**FIGURE 5:**
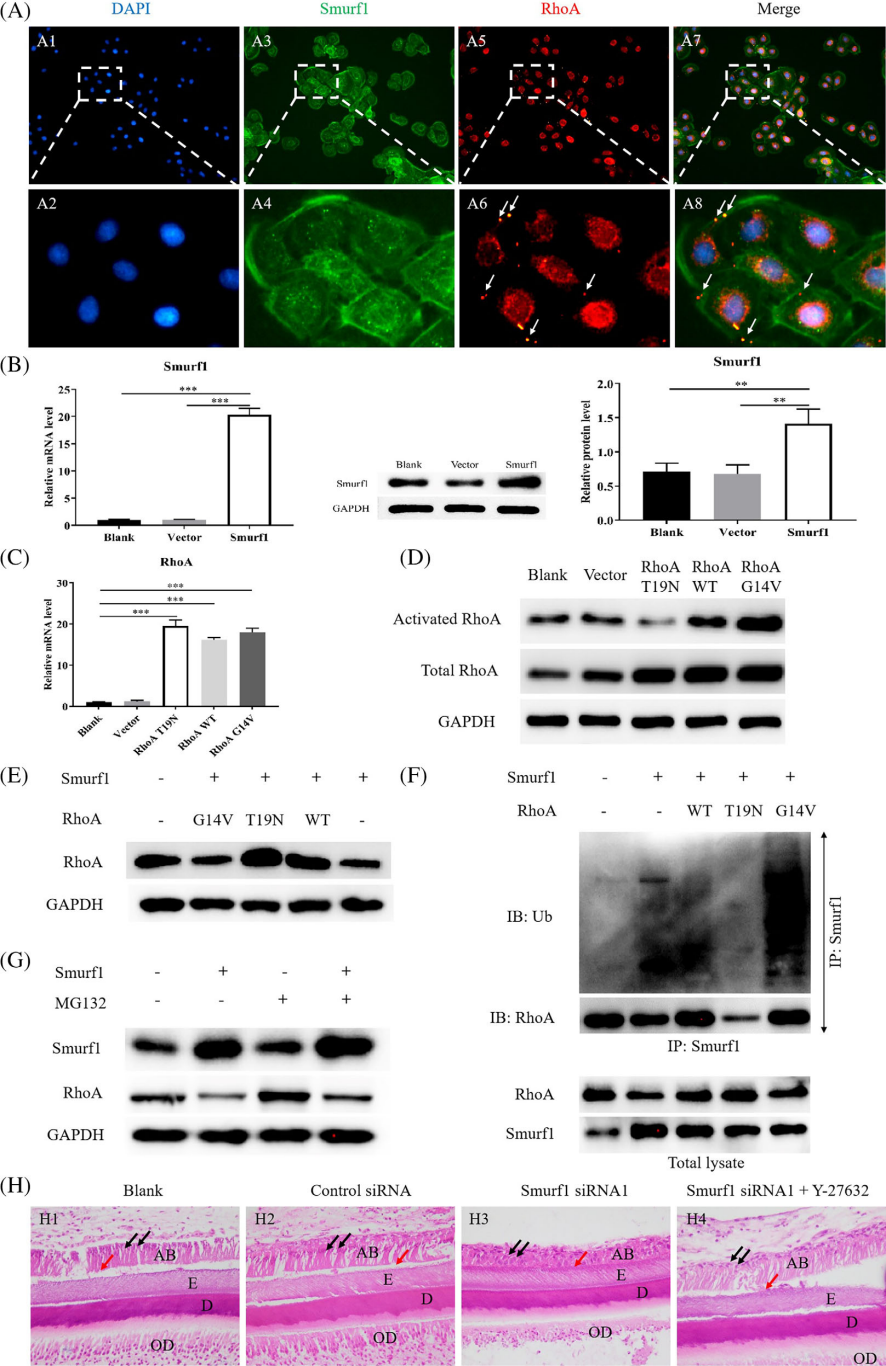
The mechanism of Smurf1 affecting ameloblast polarization by ubiquitination‐mediated degradation of RhoA. (A) Immuno‐fluorescence of Smurf1 and RhoA in HAT‐7 cells [magnification: A1, A3, A5, A7, 100×; A2, A4, A6, A8, 400×]. (B) Gene expression detection of Smurf1 of HAT‐7 cells after treated with blank, vector and Smurf1 overexpression plasmid by RT‐qPCR; Protein expression detection of Smurf1 of HAT‐7 cells after treated with blank, vector and Smurf1 overexpression plasmid by western blot and quantitative analysis. (C) Gene expression detection of RhoA of HAT‐7 cells after treated with blank, vector, RhoA T19N(inhibitory), RhoA G14V(activation) and RhoA WT(wild type) plasmid by RT‐qPCR. (D) Protein expression detection of activated and total RhoA of HAT‐7 cells after treated with blank, vector, RhoA T19N(inhibitory), RhoA G14V(activation) and RhoA WT(wild type) plasmid by western blot. (E) Protein expression detection of RhoA of HAT‐7 cells after treated with Smurf1 and different RhoA overexpression plasmids by western blot. (F) Protein Co‐IP experiment of confirming the interaction among Smurf1, RhoA and Ubiquitin. (G) Protein expression detection of Smurf1 and RhoA to verify whether Smurf1 regulated RhoA expression through ubiquitination of HAT‐7 cells after treated with proteasome inhibitor MG132. (H) H&E staining of lower incisors of postnatal 5–7 days SD rat cultured in a 3D in vitro environment of trans‐well membrane system with blank, control siRNA, Smurf1 siRNA1 and Smurf1 siRNA1 + Y‐27632 [magnification: 400×]

In Figure 5A, IF double staining assay exhibited that Smurf1 was mainly expressed in the membrane and nucleus, and was also positively expressed in the perinuclear cytoplasm. RhoA was strongly expressed in the nuclear membrane and nucleus region, and showed highly positive punctate expression on the cell membrane. The activated RhoA which Smurf1 was mainly responsible for degrading^34^, ^37^ were primarily located in the membrane while the inactive RhoA was located in the cytoplasm and nucleus. These results indicated that Smurf1 and activated RhoA co‐located on the membrane, suggesting that there might be an interaction between them.

Compared with the blank group, the expression level of Smurf1 in the overexpression group was testified to be increased by 20.3 times via RT‐qPCR and 1.98 times via western blot (Figure 5B, *p* < 0.05). RT‐qPCR showed that RhoA expression significantly increased in RhoA T19N, RhoA WT and RhoA G14V groups compared with the blank and the vector group (Figure 5C, *p* < 0.05). Western Blot results displayed the same trend of total RhoA expression (Figure 5D). However, the expression level of activated RhoA was lowered in RhoA T19N group, slightly raised in RhoA WT group, and significantly elevated in RhoA G14V group (Figure 5D). The above results suggested that the overexpression efficiency of Smurf1 and RhoA was high and the HAT‐7 cells transfected with the plasmids could be used for further study.

Smurf1 and different activated status‐RhoA overexpression plasmids were transfected in HAT‐7 cells simultaneously. RhoA T19N could have the inactivated RhoA expression increased while RhoA G14V could have the activated RhoA expression increased. RhoA expression in Smurf1+/RhoA− group decreased compared with Smurf1−/RhoA− group due to Smurf1 overexpression. RhoA expression in Smurf1+/RhoA WT group was close to that of Smurf1−/RhoA− group, but increased compared with that of Smurf1+/RhoA− group. RhoA expression in Smurf1+/RhoA T19N group was higher than the other groups. RhoA expression in Smurf1+/RhoA G14V was close to that of Smurf1+/RhoA− group (Figure 5E). These results indicated that overexpression of Smurf1 could inhibit the expression of RhoA. RhoA WT or RhoA T19N could reverse the inhibitory effect while RhoA G14V could not. These results also suggested that Smurf1 could inhibit the expression of RhoA in activated state.

Smurf1 and RhoA could be detected in the whole cell lysates of all groups, suggesting that the whole cell lysates could be used for subsequent Co‐IP experiments. When Smurf1 antibody was used to pull down the protein complex, RhoA expression was detected in the control group (Smurf1−/RhoA−), while Ub expression was low. In Smurf1+/RhoA− group, RhoA expression decreased, yet Ub expression increased compared with the control group. In Smurf1+/RhoA WT group, RhoA and Ub expression increased compared to those in the control group and Smurf1+/RhoA− group. However, in Smurf1+/RhoA T19N group, RhoA expression significantly decreased. In Smurf1+/RhoA G14V group, Ub expression level significantly elevated compared to the other groups (Figure 5F). The above results indicated that Smurf1 had a direct effect on activated RhoA and Ub.

To further verify whether Smurf1 regulated the expression of RhoA through ubiquitination, we adopted the proteasome inhibitor MG132 to inhibit the function of the UPS. The expression of RhoA was significantly down‐regulated in Smurf1 overexpression group, and MG132 could reverse the changes (Figure 5G). Combined with the above results, Smurf1 degraded RhoA through ubiquitination in AB.

To testify that Smurf1 regulate cell polarity via degradation of RhoA, in vitro 3D culture of SD rat's mandibular incisor with trans‐well system had been employed. In the blank group, the negative control group and the rescue group, AB presented high columnar shape, palisade arrangement and the nuclei being far away from the basement membrane (Figure 5H, H1, H2, H4). However, in the Smurf1 siRNA1 group, the AB were disordered and the polarity structure was damaged (Figure 5H, H3). These results demonstrated that Smurf1 regulated ameloblast polarity through ubiquitination degradation of RhoA in vitro.

### 3.5 The in vivo study of Smurf1 regulating ameloblast polarization

**FIGURE 6:**
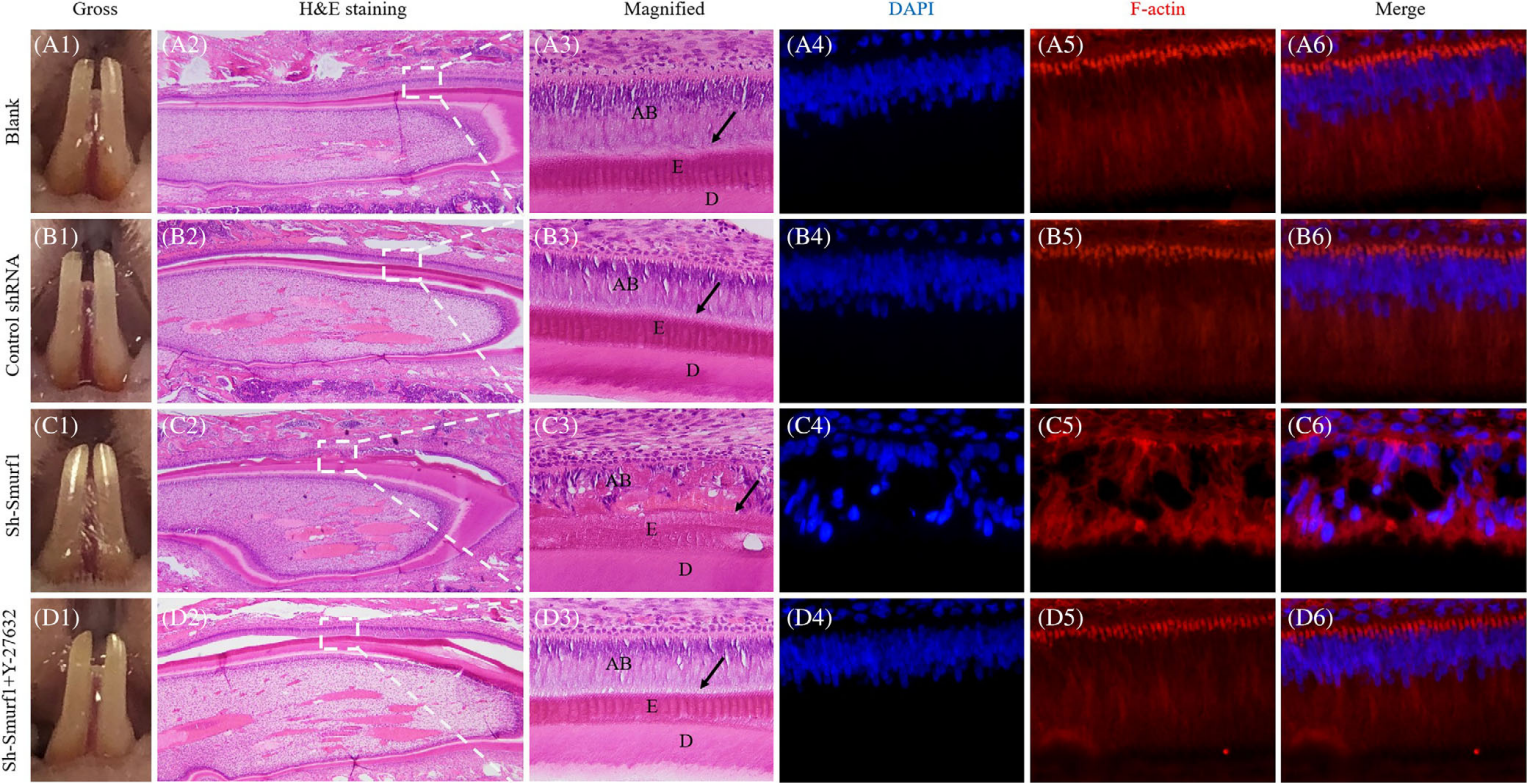
The in vivo study of Smurf1 regulating ameloblast polarization. General observation of the rat's mandibular incisors treated with blank, control shRNA, sh‐Smurf1 and sh‐Smurf1 + Y‐27632 injection (A1, B1, C1, D1); Microscopic detection of the rat's mandibular incisors treated with blank, control shRNA, sh‐Smurf1 and sh‐Smurf1 + Y‐27632 injection by H&E staining (A2, A3, B2, B3, C2, C3, D2, D3) [magnification: A2, B2, C2, D2, 40×; A3, B3, C3, D3, 400×]; Immuno‐fluorescence detection of the rat's mandibular incisors treated with blank, control shRNA, sh‐Smurf1 and sh‐Smurf1 + Y‐27632 injection (A4, A5, A6, B4, B5, B6, C4, C5, C6, D4, D5, D6) [magnification: 400×]

Rats were divided into four groups then injected respectively with normal saline, lentivirus packed with control shRNA, lentivirus packed with sh‐Smurf1 and lentivirus packed with the mixture of sh‐Smurf1 and Y‐27632. In the blank group, the vector group and the mixture group, the enamel was transparent and displayed faint yellow with a shiny surface (Figure 6, A1, B1, D1). But in the sh‐Smurf1 group, the enamel exhibited chalky, and the original brown‐yellow lustre disappeared (Figure 6, C1).

For H&E staining, the AB in the blank, vector and mixture group were arrayed in high columnar shape, with palisade arranged nuclei far away from the basement membrane. Besides, the secreted enamel matrix exhibited a regular structure and uniform thickness, siting neatly between AB and dentin (Figure 6, A2, A3, B2, B3, D2, D3). However, the AB in the sh‐Smurf1 group appeared to lost polarity showing irregular cell arrangement and tanglesome distribution of nuclei. Part of the secreted matrix was in a chaotic direction, and distributed between the AB in irregular shapes (Figure 6, C2, C3). The IF staining images showed that compared with blank, negative control and rescue groups, AB in Sh‐Smurf1 group presented irregular nuclei positions, significantly changed cytoskeletal, loss of cell polarity and blank spaces observed among disordered AB (Figure 6, A6, B6, C6, D6). These results suggested that Smurf1 knockdown could affect the polarity of AB and enamel formation in vivo, but RhoA inhibitor Y‐27632 could reverse this effect.

## 4 DISCUSSION

Smurf1 expresses in a lot of organs from embryo stage to mature individual, and plays a key role in many physiological functions of the body, such as the regulation of bone development and bone homeostasis, cell proliferation and apoptosis, cell polarity, cell migration, autophagy, immune response, and so forth.^38^, ^39^ Wang et al. found that Smurf1 regulated cell polarity and protrusive activity, and was required to maintain the transformed morphology and motility of a tumour cell.^40^ However, so far, the role of Smurf1 has not been studied in enamel development. To detect whether Smurf1 expresses in ameloblast, the slices of tooth germ and lower incisor of mouse were stained with IF. We found that Smurf1, similar to F‐actin, mainly located in ABs' membrane and cell cortex during the whole process of enamel organ development. In order to quantitatively detect the expression changes of Smurf1 during AB differentiation in the lower incisor of mouse, the dental epitheliums in different regions were isolated into CL group, PAB group and AB group and the expression of Smurf1 was measured by RT‐qPCR. Our data showed that with the polarity formation and differentiation maturation of ameloblast, the expression of Smurf1 was up‐regulated, which suggested a correlation between Smurf1 and ameloblast polarization and development. In our study, the IF results showed that Smurf1 co‐localized with F‐actin, which is known to provide mechanical support for cells and control the cell morphology and cell migration through the rapid assembly and disassembly of the actin‐network,^41^ suggesting that Smurf1 might be able to interact with F‐actin and play a role in cytoskeleton and cell morphology regulation.

There is a stem cell niche in labial CL of mouse's lower incisor, which renders the tooth continuing eruption for lifetime and repair after injury.^42^ In our study, the formation of new enamel and the growth of the damaged mouse lower incisor were observed 3 days after injury, further confirming the existence and function of cervical stem cell niche. We found that 3 days after injury, the expression of Smurf1 in CL group and AB group was not significantly different from that in the control group, while the expression of Smurf1 in PAB group was significantly increased. The possible reason was that in the injured lower incisor, the cells in CL region were in the stage of rapid division and proliferation. The cells in AB region were in the stage of terminal differentiation, during which they were able to continuously secrete enamel matrix proteins. The cells in both CL and AB regions had no evident alteration of cytoskeletal and cell morphology. However, the cells in PAB region were in the active stage of polarity formation and differentiation during which the cytoskeleton was rapidly adjusted. Previous studies have shown that Smurf1 plays an important role in cytoskeletal adjustment and polarity formation.^37^, ^43^ So, we concluded that Smurf1 might be mainly involved in the differentiation and maturation of cubic or short columnar pre‐AB to high columnar AB during the repair of injured lower incisors, and therefore its expression in the PAB group was significantly increased. In addition, during injury repair, the expression of Smurf1 in PAB group was higher than that in AB group, suggesting that the expression of Smurf1 in the process of injury repair was different from that in normal tooth development. The specific reasons need to be explored by further studies.

After knocking down Smurf1 in ameloblast in vitro, not only the expression level of Smurf1 was decreased, but also the expression location of Smurf1 and the ameloblast morphology changed obviously. In previous studies, Smurf1 has been shown to modulate a series of molecules including RhoA, Rap1, talin head and Hpem‐2, which regulate lamellipodium protrusions, cell adhesion, cell migration and cell polarity by controlling actin reorganization.^44^, ^45^ The abnormal function of Smurf1 is the pathogenic basis of many diseases, such as bone and cartilage lesions, the occurrence and development of cancer, cardiovascular diseases, inflammatory diseases, neurodegeneration and so on.^27^, ^35^, ^40^, ^46^, ^47^ Our study found that in HAT‐7 cells, Smurf1 mostly expressed in the membrane and nuclei, with a little in the cytoplasm. But, in Smurf1 knockdown AB, Smurf1 mainly located in the cytoplasm around the nuclei. It might be caused by the change of cell morphology and the reduction of cell protrusion after Smurf1 knockdown. To further identify this, the cell morphology of AB was detected by SEM. We found that after knocking down Smurf1, AB shrinked and the majority of microvilli disappeared, which further testified that Smurf1 could regulate AB's cytoskeletal and cell morphology.

The polarity of ameloblast is essential for the secretion of enamel matrix and tooth development. Zhang et, al. discovered that in Satb1(−/−) mice, the apical architecture of ameloblast was deformed, and the transport of AMGN to the apical secretory front and enamel space was impeded, resulting in a massive cytoplasmic accumulation of AMGN and a thin layer of hypo‐mineralized enamel.^48^ In Otsu's study, the polarity of ameloblast was perturbed, resulting in the disruption of vectorial expression of AMGN in RhoA (−/−) mice.^32^ In our study, after inhibiting the expression of Smurf1 by culturing SD rats' tooth germs in vitro and in vivo local injection of SD rats' lower incisors, the AB lost polarity, the secreted enamel matrix was in disorder and the gross observation displayed chalky and opaque enamel, which identified that Smurf1 was essential for ameloblast polarity and enamel development.

Smurf1 also produces an influence on cell differentiation, cell adhesion and cell migration. For example, Smurf1 was required for the myogenic differentiation of C2C12 cells and played an important regulatory role in the BMP‐2‐mediated osteoblast conversion.^49^ In our study, the differentiation of Smurf1 knockdown ameloblast was inhibited, showing decreased AMGN, AGBN, MMP20 and KLK4 expression. Besides, the migration of Smurf1 knocking down ameloblast was suppressed. Similar to our results, in the developing cerebellar cortex, the acute knockdown of Smurf1 impaired the elongation and migration of axon.^50^ In breast cancer, overexpressed Smurf1 resulted in disrupted F‐actin cytoskeletal organization, reduced cell adhesion, increased cell migration and invasion, and promoted tumour metastasis.^51^ All these indicated that Smurf1 played a role in ameloblast differentiation and migration.

In the IF experiment, Smurf1 and RhoA were co‐localized in the membrane. In Wang's study, Smurf1 was localized to lamellipodia, namely the protuberance of the cell, where it targeted RhoA for ubiquitination and degradation, thus inhibiting stress fibre formation to facilitate protrusive activity, cell migration and cell polarity.^40^ Smurf1 was also discovered to function as a substrate switch by selectively degradation of RhoA to control their localization and thereby regulated neuronal polarity.^21^ RhoA T19N plasmid could have the inactivated RhoA expression increased while RhoA G14V plasmid could have the activated RhoA expression increased. We found that the expression of RhoA was decreased in Smurf1 overexpressed ameloblast, which could be reversed by the overexpression of RhoA T19N and RhoA WT, but could not be reversed by RhoA G14V. The Co‐IP experiment showed that the Ub pulled down by Smurf1 increased significantly in the group simultaneously overexpressing Smurf1 and RhoA G14V, increased slightly in the group only overexpressing Smurf1 and simultaneously overexpressing Smurf1 and RhoA WT, but decreased in the group simultaneously overexpressing Smurf1 and RhoA T19N. The decreased expression of RhoA due to Smurf1 overexpression could be rescued by proteasome inhibitor MG132. In Boyer's study, Ub‐mediated proteasomal degradation of activated RhoA was impaired in Smurf1‐deficient cells,^52^ which were consistent with our study. In addition, Tian further found that Smurf1 bound with and ubiquitination degraded RhoA through the C2 domain.^19^ The results of our in vitro tooth germ culture and in vivo animal model showed that Smurf1 knocking down could influence ameloblast's cell polarity, enamel matrix secretion and tooth development. RhoA inhibitor Y‐27632 could reverse the above described effects. Based on all these, we identified that Smurf1 ubiquitination degraded activated RhoA to control ameloblast polarity.

In our study, after Smurf1 knockdown, RhoA expression was upregulated and the polarity of AB was inhibited. Yet, Otsu et al.^32^ had claimed a contrary opinion that down‐regulated expression of RhoA led to the damage of AB' polarity. We speculated that RhoA expression level should be maintained in an appropriate range in order to keep normal development of enamel. The expression level of RhoA either too high or too low would not benefit cells' differentiation or polarity formation. Zhu et al.^53^ found that low levels of constitutively active RhoA expression associated with segmental foot‐process effacement without changes observable by light microscopy, whereas higher levels of constitutively active RhoA expression associated with both extensive foot‐process effacement and histologic features of focal segmental glomerulosclerosis. So, proper range of RhoA is essential to maintain AB polarity and form enamel, which is worth to be studied in future work.

Smurf1 has also been found to play an important role in regulating dental pulp stem cells (DPSCs) differentiation and odontoblasts' differentiation and homeostasis. Yang et al.^54^ found that the inhibition of Smurf1 in DPSCs significantly increased RUNX2 at the protein level which was associated with Smurf1 level during odontoblastic differentiation. The knockdown of Smurf1 significantly up‐regulated RUNX2 expression and down‐regulated dentin sialophosphoprotein and dental matrix protein‐1 expression in odontoblastic differentiation. Lee et al.^55^ found that Smurf1 and Smurf2 induced NFI‐C degradation and polyubiquitination in a TGF‐β1‐dependent manner and NFI‐C was significantly degraded after TGF‐β1 addition in odontoblasts. However, there is still a blank of in vivo verification of Smurf1's role on odontoblasts and dentin formation which is worth further exploring.

In our future study, we are dedicating ourselves to conduct further research on the upstream signalling pathway of Smurf1 to form a complete and thorough mechanism for better understanding of the ameloblast polarity regulation axis. We also intend to explore the role of epithelial mesenchymal interaction (EMI) in the polarity formation of AB and odontoblasts using rodent lower incisors as an animal model, and try to extend our findings to other human organs and tissues which resemble the development pattern.

## 5 CONCLUSION

Collectively, we demonstrated that Smurf1 knocking down affected the polarization and differentiation of ameloblast and enamel development by ubiquitination degradation of activated RhoA. These findings contributed to the knowledge of understanding the regulation mechanism of ameloblast polarity, and provided new research ideas for the study of tooth development.

## AUTHOR CONTRIBUTIONS

Haoman Niu and Fei Bi performed the experiments, analysed the data and composed the manuscript. Wenjun Zhao and Yuchan Xu performed the experiments. Qi Han analysed the data. Weihua Guo and Yu Chen conceived the idea, supervised the whole progress of the research and revised the manuscript. All authors read and approved the final manuscript.

## FUNDING INFORMATION

This study was supported by the Nature Science Foundation of China (31971281).

## CONFLICT OF INTEREST

The authors declare that they have no conflict of interest.

## Supporting information


**Figure S1.** (A) Dissection and acquisition of the three groups of dental epitheliums under steoro‐microscope: cervical loop (CL), pre‐ameloblast (PAB), ameloblast (AB), (B) Typical structure of embryonic developing tooth germ: bud stage (B1), cap stage (B2), bell stage (B3), (C) Normal structure of adult mice’ mandibular incisor (C1): cervical loop (C2), pre‐ameloblast (C3), ameloblast (C4), and (D) In vitro 3D culture of PN 5‐7d SD rats' mandibular incisors with trans‐well system. DF, Dental follicle; DP, Dental papilla; E, Enamel organ; IEE, Inner enamel epithelium; M, Mesenchyme; OEE, Outer enamel epithelium; SI, Strata intermedium; SR, Stellate reticulum

## Data Availability

The datasets supporting the conclusions of this article are included within the article.

## References

[1] Lacruz RS , Habelitz S , Wright JT , Paine ML . Dental enamel formation and implications for oral health and disease. Physiol Rev. 2017;97:939‐993. 28468833 10.1152/physrev.00030.2016 PMC6151498

[2] Bei M . Molecular genetics of ameloblast cell lineage. J Exp Zool B Mol Dev Evol. 2009;312B:437‐444. 19090561 10.1002/jez.b.21261 PMC2737325

[3] Pieczynski J , Margolis B . Protein complexes that control renal epithelial polarity. Am J Physiol Renal Physiol. 2011;300:F589‐F601. 21228104 10.1152/ajprenal.00615.2010 PMC3064137

[4] Zhou Y , Ji H , Xu Q , et al. Congenital biliary atresia is correlated with disrupted cell junctions and polarity caused by Cdc42 insufficiency in the liver. Theranostics. 2021;11:7262‐7275. 34158849 10.7150/thno.49116 PMC8210598

[5] Szymaniak AD , Mahoney JE , Cardoso WV , Varelas X . Crumbs3‐mediated polarity directs airway epithelial cell fate through the hippo pathway effector yap. Dev Cell. 2015;34:283‐296. 26235047 10.1016/j.devcel.2015.06.020 PMC4536126

[6] Ridley AJ , Schwartz MA , Burridge K , et al. Cell migration: integrating signals from front to back. Science. 2003;302:1704‐1709. 14657486 10.1126/science.1092053

[7] Inaba M , Venkei ZG , Yamashita YM . The polarity protein Baz forms a platform for the centrosome orientation during asymmetric stem cell division in the drosophila male germline. Elife. 2015;4:4. 10.7554/eLife.04960 PMC4391501 25793442

[8] Hernandez P , Tirnauer JS . Tumor suppressor interactions with microtubules: keeping cell polarity and cell division on track. Dis Model Mech. 2010;3:304‐315. 20427559 10.1242/dmm.004507

[9] Wang LT , Rajah A , Brown CM , McCaffrey L . CD13 orients the apical‐basal polarity axis necessary for lumen formation. Nat Commun. 2021;12:4697. 34349123 10.1038/s41467-021-24993-x PMC8338993

[10] Martin E , Girardello R , Dittmar G , Ludwig A . New insights into the organization and regulation of the apical polarity network in mammalian epithelial cells. FEBS J. 2021;288:7073‐7095. 33448150 10.1111/febs.15710

[11] Saraswathy VM , Kurup AJ , Sharma P , Poles S , Poulain M , Furthauer M . The E3 ubiquitin ligase mindbomb1 controls planar cell polarity‐dependent convergent extension movements during zebrafish gastrulation. Elife. 2022;11:11. 10.7554/eLife.71928 PMC8937233 35142609

[12] Wang L , Bu T , Li L , et al. Planar cell polarity (PCP) proteins support spermatogenesis through cytoskeletal organization in the testis. Semin Cell Dev Biol. 2022;121:99‐113. 34059418 10.1016/j.semcdb.2021.04.008

[13] Pena A , Ouarne M , Franco CA . Methods to quantify endothelial cell front‐rear polarity in vivo and in vitro. Curr Opin Hematol. 2021;28:208‐213. 33656462 10.1097/MOH.0000000000000643

[14] Saito K , Mori M , Kambara N , Ohta Y . FilGAP, a GAP protein for Rac, regulates front‐rear polarity and tumor cell migration through the ECM. FASEB J. 2021;35:e21508. 33710706 10.1096/fj.202002155R

[15] Nastaly P , Purushothaman D , Marchesi S , et al. Role of the nuclear membrane protein Emerin in front‐rear polarity of the nucleus. Nat Commun. 2020;11:2122. 32358486 10.1038/s41467-020-15910-9 PMC7195445

[16] Nir I , Amador G , Gong Y , et al. Evolution of polarity protein BASL and the capacity for stomatal lineage asymmetric divisions. Curr Biol. 2022;32:329‐337. 34847354 10.1016/j.cub.2021.11.013

[17] Sun Z , Tang Y , Zhang Y , et al. Joint single‐cell multiomic analysis in Wnt3a induced asymmetric stem cell division. Nat Commun. 2021;12:5941. 34642323 10.1038/s41467-021-26203-0 PMC8511096

[18] Sardi J , Bener MB , Simao T , Descoteaux AE , Slepchenko BM , Inaba M . Mad dephosphorylation at the nuclear pore is essential for asymmetric stem cell division. Proc Natl Acad Sci USA. 2021;118(13):e2006786118. 10.1073/pnas.2006786118 PMC8020777 33753475

[19] Tian M , Bai C , Lin Q , et al. Binding of RhoA by the C2 domain of E3 ligase Smurf1 is essential for Smurf1‐regulated RhoA ubiquitination and cell protrusive activity. FEBS Lett. 2011;585:2199‐2204. 21708152 10.1016/j.febslet.2011.06.016

[20] Lu K , Li P , Zhang M , et al. Pivotal role of the C2 domain of the Smurf1 ubiquitin ligase in substrate selection. J Biol Chem. 2011;286:16861‐16870. 21402695 10.1074/jbc.M110.211979 PMC3089529

[21] Stiess M , Bradke F . Controlled demolition: Smurf1 regulates neuronal polarity by substrate switching. Neuron. 2011;69:183‐185. 21262456 10.1016/j.neuron.2011.01.007

[22] Critchley DR . Smurf1 zaps the Talin head. Nat Cell Biol. 2009;11:538‐540. 19404335 10.1038/ncb0509-538

[23] Huang C , Rajfur Z , Yousefi N , Chen Z , Jacobson K , Ginsberg MH . Talin phosphorylation by Cdk5 regulates Smurf1‐mediated Talin head ubiquitylation and cell migration. Nat Cell Biol. 2009;11:624‐630. 19363486 10.1038/ncb1868 PMC2714540

[24] Wei X , Wang X , Zhan J , et al. Smurf1 inhibits integrin activation by controlling Kindlin‐2 ubiquitination and degradation. J Cell Biol. 2017;216:1455‐1471. 28408404 10.1083/jcb.201609073 PMC5412569

[25] Bryan B , Cai Y , Wrighton K , Wu G , Feng XH , Liu M . Ubiquitination of RhoA by Smurf1 promotes neurite outgrowth. FEBS Lett. 2005;579:1015‐1019. 15710384 10.1016/j.febslet.2004.12.074

[26] Garcia‐Garcia P , Ruiz M , Reyes R , et al. Smurf1 silencing using a LNA‐ASOs/lipid nanoparticle system to promote bone regeneration. Stem Cells Transl Med. 2019;8:1306‐1317. 31631568 10.1002/sctm.19-0145 PMC6877774

[27] Yamashita M , Ying SX , Zhang GM , et al. Ubiquitin ligase Smurf1 controls osteoblast activity and bone homeostasis by targeting MEKK2 for degradation. Cell. 2005;121:101‐113. 15820682 10.1016/j.cell.2005.01.035 PMC3314294

[28] Feng X , Jia Y , Zhang Y , et al. Ubiquitination of UVRAG by SMURF1 promotes autophagosome maturation and inhibits hepatocellular carcinoma growth. Autophagy. 2019;15:1130‐1149. 30686098 10.1080/15548627.2019.1570063 PMC6613838

[29] Zhu M , Cornwall‐Scoones J , Wang P , et al. Developmental clock and mechanism of de novo polarization of the mouse embryo. Sci. 2020;370:370. 10.1126/science.abd2703 PMC8210885 33303584

[30] Etienne‐Manneville S , Hall A . Rho GTPases in cell biology. Nature. 2002;420:629‐635. 12478284 10.1038/nature01148

[31] O'Connor K , Chen M . Dynamic functions of RhoA in tumor cell migration and invasion. Small GTPases. 2013;4:141‐147. 24025634 10.4161/sgtp.25131 PMC3976970

[32] Otsu K , Ida‐Yonemochi H , Fujiwara N , Harada H . The Semaphorin 4D‐RhoA‐Akt signal Cascade regulates enamel matrix secretion in coordination with cell polarization during Ameloblast differentiation. J Bone Miner Res. 2016;31:1943‐1954. 27218883 10.1002/jbmr.2876

[33] Cho HJ , Kim JT , Baek KE , Kim BY , Lee HG . Regulation of rho GTPases by RhoGDIs in human cancers. Cells‐Basel. 2019;8:8. 10.3390/cells8091037 PMC6769525 31492019

[34] Wei J , Mialki RK , Dong S , et al. A new mechanism of RhoA ubiquitination and degradation: roles of SCF(FBXL19) E3 ligase and Erk2. Biochim Biophys Acta. 2013;1833:2757‐2764. 23871831 10.1016/j.bbamcr.2013.07.005 PMC3834026

[35] Lee MG , Jeong SI , Ko KP , et al. RASSF1A directly antagonizes RhoA activity through the assembly of a Smurf1‐mediated destruction complex to suppress tumorigenesis. Cancer Res. 2016;76:1847‐1859. 26825171 10.1158/0008-5472.CAN-15-1752

[36] Yu F , Li F , Zheng L , Ye L . Epigenetic controls of sonic hedgehog guarantee fidelity of epithelial adult stem cells trajectory in regeneration. Sci Adv. 2022;8:n4977. 10.1126/sciadv.abn4977 PMC9307244 35867784

[37] Ozdamar B , Bose R , Barrios‐Rodiles M , Wang HR , Zhang Y , Wrana JL . Regulation of the polarity protein Par6 by TGFbeta receptors controls epithelial cell plasticity. Sci. 2005;307:1603‐1609. 10.1126/science.1105718 15761148

[38] Li H , Xiao N , Wang Y , et al. Smurf1 regulates lung cancer cell growth and migration through interaction with and ubiquitination of PIPKIgamma. Oncogene. 2017;36:5668‐5680. 28581524 10.1038/onc.2017.166

[39] Sanchez NS , Barnett JV . TGFbeta and BMP‐2 regulate epicardial cell invasion via TGFbetaR3 activation of the Par6/Smurf1/RhoA pathway. Cell Signal. 2012;24:539‐548. 22033038 10.1016/j.cellsig.2011.10.006 PMC3237859

[40] Wang HR , Zhang Y , Ozdamar B , et al. Regulation of cell polarity and protrusion formation by targeting RhoA for degradation. Science. 2003;302:1775‐1779. 14657501 10.1126/science.1090772

[41] Belyy A , Merino F , Sitsel O , Raunser S . Structure of the Lifeact‐F‐Actin complex. PLoS Biol. 2020;18:e3000925. 33216759 10.1371/journal.pbio.3000925 PMC7717565

[42] Boran T , Peterkova R , Lesot H , Lyons DB , Peterka M , Klein OD . Temporal analysis of ectopic enamel production in incisors from sprouty mutant mice. J Exp Zool B Mol Dev Evol. 2009;312B:473‐485. 19101957 10.1002/jez.b.21254 PMC2837846

[43] Hunter KW . Ezrin, a key component in tumor metastasis. Trends Mol Med. 2004;10:201‐204. 15121044 10.1016/j.molmed.2004.03.001

[44] Deng S , Huang C . E3 ubiquitin ligases in regulating stress fiber, lamellipodium, and focal adhesion dynamics. Cell Adh Migr. 2014;8:49‐54. 24589622 10.4161/cam.27480 PMC3974793

[45] Huang C . Roles of E3 ubiquitin ligases in cell adhesion and migration. Cell Adh Migr. 2010;4:10‐18. 20009572 10.4161/cam.4.1.9834 PMC2852552

[46] Koefoed K , Skat‐Rordam J , Andersen P , et al. The E3 ubiquitin ligase SMURF1 regulates cell‐fate specification and outflow tract septation during mammalian heart development. Sci Rep. 2018;8:9542. 29934521 10.1038/s41598-018-27854-8 PMC6015040

[47] Vohra BP , Fu M , Heuckeroth RO . Protein kinase Czeta and glycogen synthase kinase‐3beta control neuronal polarity in developing rodent enteric neurons, whereas SMAD specific E3 ubiquitin protein ligase 1 promotes neurite growth but does not influence polarity. J Neurosci. 2007;27:9458‐9468. 17728459 10.1523/JNEUROSCI.0870-07.2007 PMC2267823

[48] Zhang Y , Zheng L , Le M , et al. SATB1 establishes ameloblast cell polarity and regulates directional amelogenin secretion for enamel formation. BMC Biol. 2019;17:104. 31830989 10.1186/s12915-019-0722-9 PMC6909472

[49] Ying SX , Hussain ZJ , Zhang YE . Smurf1 facilitates myogenic differentiation and antagonizes the bone morphogenetic protein‐2‐induced osteoblast conversion by targeting Smad5 for degradation. J Biol Chem. 2003;278:39029‐39036. 12871975 10.1074/jbc.M301193200 PMC3230132

[50] Kannan M , Lee SJ , Schwedhelm‐Domeyer N , Stegmuller J . The E3 ligase Cdh1‐anaphase promoting complex operates upstream of the E3 ligase Smurf1 in the control of axon growth. Development. 2012;139:3600‐3612. 22949615 10.1242/dev.081786

[51] Yu L , Liu X , Cui K , et al. SND1 acts downstream of TGFbeta1 and upstream of Smurf1 to promote breast cancer metastasis. Cancer Res. 2015;75:1275‐1286. 25596283 10.1158/0008-5472.CAN-14-2387

[52] Boyer L , Turchi L , Desnues B , et al. CNF1‐induced ubiquitylation and proteasome destruction of activated RhoA is impaired in Smurf1−/− cells. Mol Biol Cell. 2006;17:2489‐2497. 16540523 10.1091/mbc.E05-09-0876 PMC1474842

[53] Zhu L , Jiang R , Aoudjit L , Jones N , Takano T . Activation of RhoA in podocytes induces focal segmental glomerulosclerosis. J Am Soc Nephrol. 2011;22:1621‐1630. 21804090 10.1681/ASN.2010111146 PMC3171934

[54] Yang F , Xu N , Li D , et al. A feedback loop between RUNX2 and the E3 ligase SMURF1 in regulation of differentiation of human dental pulp stem cells. J Endod. 2014;40:1579‐1586. 25260729 10.1016/j.joen.2014.04.010

[55] Lee DS , Yoon WJ , Cho ES , et al. Crosstalk between nuclear factor I‐C and transforming growth factor‐beta1 signaling regulates odontoblast differentiation and homeostasis. PLoS One. 2011;6:e29160. 22195013 10.1371/journal.pone.0029160 PMC3241690

