# Supplementary figures and images for: Smurf1 regulates ameloblast polarization by ubiquitination‐mediated degradation of RhoA
Source: Cell Prolif. 2022 Dec 29;56(4):e13387. doi: 10.1111/cpr.13387 (PMC10068949; doi:10.1111/cpr.13387)

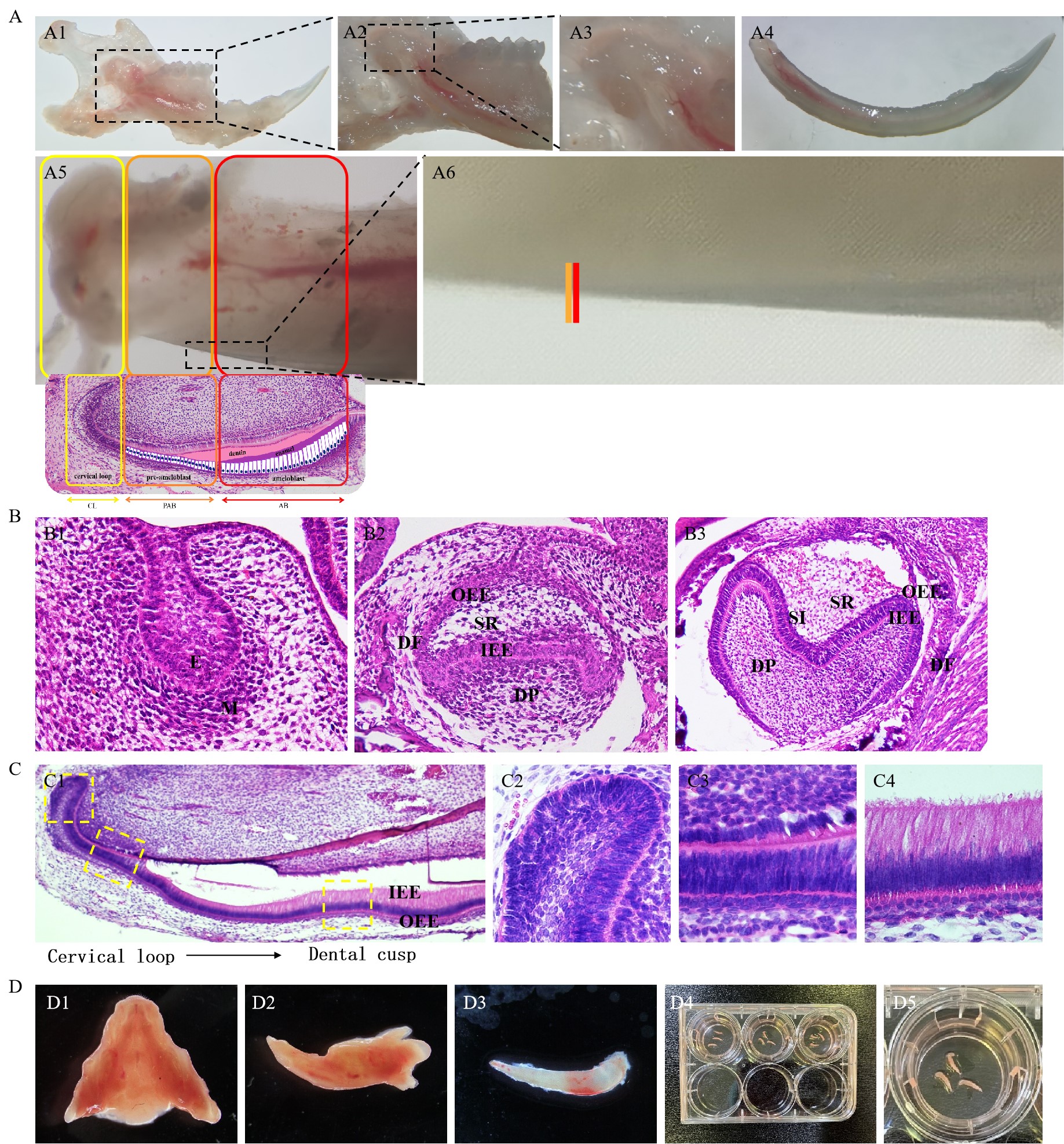

Supplement: Supplementary file 1 — Figure S1. (A) Dissection and acquisition of the three groups of dental epitheliums under steoro‐microscope: cervical loop (CL), pre‐ameloblast (PAB), ameloblast (AB), (B) Typical structure of embryonic developing tooth germ: bud stage (B1), cap stage (B2), bell stage (B3), (C) Normal structure of adult mice’ mandibular incisor (C1): cervical loop (C2), pre‐ameloblast (C3), ameloblast (C4), and (D) In vitro 3D culture of PN 5‐7d SD rats' mandibular incisors with trans‐well system. DF, Dental follicle; DP, Dental papilla; E, Enamel organ; IEE, Inner enamel epithelium; M, Mesenchyme; OEE, Outer enamel epithelium; SI, Strata intermedium; SR, Stellate reticulum [file CPR-56-e13387-s001.jpg]
